# Older Adults and Substance-Related Disorders: Trends and Associated Costs

**DOI:** 10.1155/2013/905368

**Published:** 2013-04-24

**Authors:** Daniel Rosen, Emily Heberlein, Rafael J. Engel

**Affiliations:** ^1^School of Social Work, University of Pittsburgh, 2117 Cathedral of Learning, Pittsburgh, PA 15260, USA; ^2^Arnold School of Public Health, University of South Carolina, 800 Sumter Street, HESC 112, Columbia, SC 29208, USA

## Abstract

*Purpose*. The aim of this study is to examine the changing service profile of older adults receiving substance abuse services over the past decade and the increased costs of treating this population. *Design and Methods*. Medicaid claims for mental health and substance abuse services data from a medium sized county in an eastern state were analyzed for individuals aged 50 years and older in calendar year 2000 or 2009. Univariate statistics are presented to describe the substance abuse and mental health services used by older adults in these two years. *Results*. The number of low-income older adults who accessed services for treatment and who had a substance-related diagnosis grew from 545 individuals in 2000 to 1,653 individuals in 2009. Costs for services utilized by older adults with a substance-related diagnosis rose by 358% from $2.1 million in 2000 to $9.5 million in 2009. *Implications*. The increase in the number of low-income older adults with a substance-related disorder and the concomitant rise in total spending for Medicaid reimbursed services indicate that local and state social service providers need to prepare for an older adult population who will need appropriate substance abuse prevention and treatment programs.

## 1. Introduction

Between 2006 and 2008, approximately 4.3 million adults aged 50 or older (4.7% of adults over 50) had used an illicit drug in the past year [1]. This has been accompanied by an increased prevalence of substance abuse and dependence reflected in increased alcohol and substance abuse treatment admission rates. Older adult substance abuse treatment admissions (aged 50 or older) increased from 6.6% of all admissions aged 12 years or older in 1992 to 12.2% in 2008 [2, 3]. As compared to earlier generations, a higher proportion of the baby-boom cohort used recreational drugs at a younger age, and many have entered later life with significant histories of drug and alcohol use [4]. In addition, this emerging cohort of older adults has greater access to highly addictive prescription narcotics to treat pain [5]. 

The graying of the substance abuse treatment population was accompanied by a changing profile of the primary substance that prompted older adults to enter treatment. In 1992, 84.6% of older adults entering treatment reported alcohol as their primary substance of abuse compared to just 59.9% in 2008. During this same time period, the proportion that reported primary heroin abuse more than doubled from 7.2% to 16.0% [2, 3].

While there has been a shift in the age demographics of substance abuse treatment clients in the United States, there has been little consideration of the implications of a larger older adult treatment population and the associated costs of treatment. Because the proportion of older adults 50 years of age or older in Allegheny County, PA, 37%, is higher than the national average of 30%, Allegheny County provides a case study for examining the aging demographics of the substance abuse treatment population. With a population of slightly more than a million people, an analysis of Allegheny County provides a snapshot of what the rest of the country will face in the coming decades as the baby-boom generation swells the ranks of older adults in the United States. This paper examines county level administrative data in Allegheny County, Pennsylvania, to illustrate the unique characteristics of an emerging group of low-income older adults utilizing substance abuse treatment. Specifically, we present data on the changing service profile of older adults receiving substance abuse services over the past decade and the increased costs of treating this population. 

## 2. Design and Methods

### 2.1. Sample

This analysis is drawn from Medicaid claims data in Allegheny County, Pennsylvania, for individuals over 50 who used substance abuse treatment services in calendar year 2000 or 2009. Paid claims were analyzed for all individuals who had received at least one service with a primary substance-related disorder submitted on the claim. All behavioral health services during each year associated with these individuals were included, whether they were mental health or substance use treatment services.

In 2000, 545 Medicaid recipients over 50 years of age received treatment for a substance-related disorder; this number grew to 1,653 individuals in 2009. The gender distribution only varied slightly; in 2000 68.81% of the cohort was male, and 66.12% was male in 2009. Over a ten-year period the racial distribution changed from a majority of African Americans (60.9%) to a majority of whites (53.1%). Those individuals identifying as Hispanic, Native American, Asian American, and other races comprised less than 2% of the cohort in each year. The average age of both cohorts was almost equivalent; the 2000 cohort ranged from 50 to 79 years old and had an average age of 55.2 (SD = 5.17) while the 2009 cohort ranged from 50 to 83 years old and had an average age of 55.0 (SD = 4.43). 

### 2.2. Analyses

Univariate statistics are presented to describe the substance abuse and mental health services used by older adults in 2000 and 2009. We also present data on the costs associated with services used by older adults with substance-related diagnoses. A determination of a primary diagnosis was made by identifying the most common substance-related diagnoses submitted on claims for a person within the given year. 

## 3. Results

In the last decade, the number of older adults who accessed services for treatment of substance-related disorders has grown. The total number of older adult participants who accessed services for treatment of and who had a substance-related diagnosis grew from 545 individuals in 2000 to 1,653 individuals in 2009 (see Table 1), a change of 203%; in contrast, the number of people 50+ who accessed services and did not have a primary substance abuse diagnosis increased from 2,861 in 2000 to 5,860 in 2009, a change of 105%. Of all Medicaid claim service users aged 50 and older, those with a primary substance-related disorder comprised 16% in 2000 and 22% in 2009. Concomitantly, costs for services utilized by older adults with a substance-related diagnosis rose 358% from $2.1 million in 2000 to $9.5 million in 2009. In 2009, the cost of services for people with substance use disorders made up 31% of all paid claims for individuals aged 50 and older versus 27% in 2000. The cost per older adult service user rose from $3,811 in 2000 to $5,771 in 2009; the nearly 50% growth in treatment cost per person was double the inflation rate between those two dates (24.6%).

Table 2 reveals that in 2000 and 2009, almost half the participants had a primary diagnosis of opiate dependence (45% and 46%) with the second most common diagnosis being an alcohol disorder (25% and 24%). Primary diagnosis of stimulants and polysubstance dependence both increased substantially; stimulants, in particular cocaine, increased from 6.8% to 10.2% (a 50% increase) while polysubstance dependence increased from 12.3% to 17.4% (change of 41%). A considerable drop was found for hallucinogens, sedatives, inhalants, and Phencyclidine** (**PCP) declining from 9.9% to 0.5% (a 94% decline). 

Table 3 identifies the different services used in 2000 and 2009 by older adults enrolled in Medicaid with substance-related diagnoses. The percentage of enrollees using all services except for inpatient mental health services, partial hospitalization services, and methadone services grew from 2000 to 2009. Of particular note is that many older adults with substance-related diagnoses also accessed mental health treatment services. In 2009, nearly 40% of older adults receiving substance abuse services also received outpatient mental health services, which was more than double the proportion who accessed such services in 2000. Over a third (37%) of the total cost of treatment for older adults with substance use disorders was for mental health services (not shown in the table). 

## 4. Discussion

The findings presented in this paper identify that the older adult population of individuals diagnosed with substance-related disorders has grown both in number and percentage of the overall older adult Medicaid service-using population between 2000 and 2009. The chronic nature of alcohol and drug disorders along with the high rates of cooccurring mental health disorders within the substance using population, as indicated by the use of mental health services, is one contributor to the higher cost of treating this population.

The trends from these data suggest that local and state governments and social service providers must prepare for an aging population of individuals who will need appropriate prevention and treatment services. Data from our analyses indicate that as more older adults receive treatment for substance use and cooccurring mental health disorders, there will also be an associated increased total cost for services.

We found a higher proportion of older adults with a primary diagnosis of opioid use (45.2%) than the national data (16%; [2, 3]). We are uncertain whether this is related to a sample difference (low income in comparison to the broader population) or how primary diagnosis was defined. Nonetheless, both the trends in the SAMHSA [2, 3] report and our findings reflect the increased need for opioid addiction treatment for older adults. Methadone maintenance treatment programs will need to address the changing demographics of this population in terms of their service delivery [6, 7]. 

Creating age appropriate services for older adults within the behavioral health system will be a critical factor in effectively treating this population. In order to better serve older adults with substance use disorders, greater attention will need to be paid to prevention, detection, early intervention, and treatment. At the same time, providers need to be trained in appropriate screening for drug and alcohol problems for older adults. Services and supports customized to meet the unique needs of the older adult population are lacking in many communities [8]. Only through education and training for providers of behavior and physical health services, older adults, families, and caregivers will the service system be able to respond to this emerging trend. Tailored programs for this population, taking into account the high rates of cooccurring physical and mental health disorders, will require targeted initiatives that focus on improved consumer education, as well as alternative approaches to screening and interventions for providers. 

Several limitations exist in utilizing administrative data to examine patterns of substance-related disorders and service utilization. The variables that are reported did not allow for assessing the quality of treatment or outcomes. This analysis assigned a primary diagnosis to individuals with substance-related disorders, where the primary diagnosis was determined by the frequency of diagnoses on approved Medicaid claims. Many people receive more than one type of substance-related diagnosis; therefore, narrowing to the most frequent diagnosis for any individual resulted in smaller diagnostic categories overall. In addition, since these data are claims based, they do not capture those individuals who had a substance-related disorder who were not receiving treatment for the disorder. Finally, the results are limited to a low-income population of Medicaid recipients.

As the baby-boom generation ages there will be an increased demand for treatment and services that meet the needs of older adults, including services related to substance-related disorders; Planning and policy level initiatives will need to incorporate changing demographic trends in order to adequately prepare for the types and scope of services needed to serve this population. Collaboration with physical health providers, area aging services, and other public assistance programs for older adults will be critical for creating an effective system of prevention, intervention, and treatment programs for older adults with substance-related disorders and the communities that support them.

## Figures and Tables

**Table 1:** Service users 50+ years old with a substance use diagnosis.

	2000	2009	% change
Number of service users aged 50+ with a primary substance use diagnosis	545	1653	203%
Number of service users aged 50+ with a different primary diagnosis	2,861	5,860	105%
Proportion of all service users 50+ years old	16%	22%	6%
Total cost (millions)	$2.1	$9.5	358%
Proportion of total cost for all service users 50+	27%	31%	4%
Average cost per person	$3,811	$5,771	51%

**Table 2:** Primary substance of abuse comparisons between 2000 and 2009 cohorts.

	2000 (*n* = 545)		2009 (*n* = 1650)∗	
	*N*	%	*N*	%
Primary diagnosis				
Stimulants (amphetamines and cocaine)	37	6.79	169	10.22
Cannabis	4	0.73	20	1.21
Opiates	246	45.14	766	46.34
Alcohol (ETOH)	137	25.14	402	24.32
Other (hallucinogens, sedatives, inhalants, PCP)	54	9.91	9	0.54
Other drug use and polysubstance dependence	67	12.29	287	17.37

∗Three individuals from the 2009 cohort were not assigned a diagnosis.

**Table 3:** Types of services accessed by older adults with substance use diagnoses that used behavioral health services.

	2000 (*n* = 545)	2009 (*n* = 1,650)∗	Percent change
Outpatient mental health	17%	39%	+129
Medication checks	24%	38%	+58
Inpatient mental health	17%	12%	−29
Crisis services	1%	12%	+1100
Service coordination	7%	11%	+57
Outpatient (substance use)	35%	53%	+51
Methadone maintenance	39%	37%	−5
Nonhospital rehabilitation	15%	19%	+27
Intensive outpatient (substance use)	3%	12%	+300
Partial hospitalization (substance use)	11%	8%	−27
Nonhospital detoxification	3%	7%	+133

∗Three individuals from the 2009 cohort were not assigned a diagnosis.

## References

[1] Substance Abuse and Mental Health Services Administration, Office of Applied Studies (2009). *An Examination of Trends in Illicit Drug Use among Adults Aged 50 to 59 in the United States*.

[2] Substance Abuse and Mental Health Services Administration, Office of Applied Studies (August 2010). *The TEDS Report: Sociodemographic Characteristics of Substance Abuse Treatment Admissions Aged 50 or Older: 1992 to 2008*.

[3] Substance Abuse and Mental Health Services Administration, Office of Applied Studies (June 2010). *The TEDS Report: Changing Substance Abuse Patterns Among Older Admissions: 1992 and 2008*.

[4] Lofwall M. R., Schuster A., Strain E. C. (2008). Changing profile of abused substances by older persons entering treatment. *The Journal of Nervous and Mental Disease*.

[5] Dowling G. J., Weiss S. R. B., Condon T. P. (2008). Drugs of abuse and the aging brain. *Neuropsychopharmacology*.

[6] Boeri M. W., Sterk C. E., Elifson K. W. (2008). Reconceptualizing early and late onset: a life course analysis of older heroin users. *The Gerontologist*.

[7] Rosen D., Smith M. L., Reynolds C. F. (2008). The prevalence of mental and physical health disorders among older methadone patients. *The American Journal of Geriatric Psychiatry*.

[8] Johnson P. B., Sung H. E. (2009). Substance abuse among aging baby boomers: health and treatment implications. *Journal of Addictions Nursing*.

